# Antibiotic resistance pattern and frequency of *cagA* and *vacA* genes in *Helicobacter pylori* strains isolated from patients in Tabriz city, Iran

**DOI:** 10.1186/s13104-021-05633-5

**Published:** 2021-05-31

**Authors:** Farnaz Rasi-Bonab, Abolfazl Jafari-Sales, Mohammad Amin Shaverdi, Tahereh Navidifar, Morteza Saki, Atosa Ghorbani, Abimbola Olumide Adekanmbi, Behboud Jafari, Sara Naebi

**Affiliations:** 1 Department of Microbiology, Marand Branch, Islamic Azad University, Marand, Iran; 2 Department of Microbiology, School of Basic Sciences, Kazerun Branch, Islamic Azad University, Kazerun, Iran; 3 Blood Transfusion Research Center, High Institute for Research and Education in Transfusion Medicine, Tehran, Iran; 4 Khuzestan Blood Transfusion Center, Abadan, Iran; 5 Shoushtar Faculty of Medical Sciences, Shoushtar, Iran; 6 Department of Microbiology, Faculty of Medicine, Ahvaz Jundishapur University of Medical Sciences, Ahvaz, Iran; 7 Student Research Committee, Ahvaz Jundishapur University of Medical Sciences, Ahvaz, Iran; 8 Environmental Microbiology and Biotechnology Laboratory, Department of Microbiology, University of Ibadan, Ibadan, Nigeria; 9 Department of Microbiology, Ahar Branch, Islamic Azad University, Ahar, Iran

**Keywords:** Antibiotic resistance, *cagA*, Clarithromycin, *Helicobacter pylori*, Iran, Metronidazole, *vacA*

## Abstract

**Objective:**

*Helicobacter pylori* is one of the most common causes of gastric infections in humans. It is estimated that approximately 50% of people around the world are infected with this bacterium. This study aimed to determine the antibiotic resistance pattern, as well as the frequency of *cagA* and *vacA* genes in *H. pylori* isolates obtained from patients in the clinical centers in Tabriz city, Iran.

**Results:**

The culture method detected 100 (45.25%) *H. pylori* isolates from 221 biopsy samples during 3 years. The results showed that 63% and 81% of the isolates were positive for *cagA* and *vacA* genes, respectively. The highest resistance of isolates was seen against metronidazole (79%) and amoxicillin (36%), respectively. Also, the isolates showed the least resistance to tetracycline (8%).

## Introduction

*Helicobacter pylori* is a spiral Gram-negative bacterium and the causative agent of chronic gastric infections in about half of the world’s population [1]. The infections caused by this bacterium mostly progress from childhood and remain chronically (lifelong) without any symptoms. Acute gastric infections, however, may be developed in some patients [2]. The gastric mucus of humans is the most significant place for the colonization of *H. pylori*, but it is rarely observed in other *Helicobacter* species [3]. In 1994, the World Health Organization (WHO) classified *H. pylori* as a Class I carcinogen and declared that its eradication could reduce the risk of stomach cancer [4]. *H. pylori* has also been recognized as a major cause of many gastrointestinal infections such as gastritis, duodenal and gastric ulcers, as well as mucosa-associated lymphoid tissue (MALT) lymphoma [1].

The virulence factors responsible for establishing the pathogenesis of *H. pylori* encompass urease, flagella, adhesins, cytotoxin-associated gene A (*cagA*), and vacuolating cytotoxin A (*vacA*) [5]. It seems likely that these virulence factors contribute to tissue damage, bacterial resistance to the host immune system, bacterial adaptation, and survival in the acidic environment of the stomach [5, 6]. Surveys have suggested that *cagA* and *vacA* genes, as two important determinants of *H. pylori* pathogenicity, are mainly involved in the damage to epithelial cells and chronic inflammation, which possibly results in elevated risk of gastric cancer [5, 6]. The *vacA* toxin produced by *H. pylori* can induce the formation of pores in the host cellular membrane, giving rise to the development of vacuoles in host cells [7]. The presence of the *cagA* gene is associated with the development of gastrointestinal ulcers and severe gastritis, as well as precancerous and cancerous lesions [8, 9]. Moreover, the *cagA*-positive strains have more ability to develop gastric cancer compared to *cagA*-negative strains [9].

Recently, the standard multiple therapies by a proton pump inhibitor or ranitidine bismuth citrate, together with clarithromycin and amoxicillin or metronidazole, have become the most effective protocol for treating *H. pylori* infections [10]. However, the emergence of the antibiotic resistant bacteria, including methicillin-resistant *Staphylococcus aureus*, carbapenem-resistant *Klebsiella pneumoniae*, extended spectrum β-lactamases (ESBLs)-producing *Enterobacteriaceae*, and *H. pylori* isolates in various countries has emerged as a global problem [11–14]. Therefore, evaluating the antibiotic resistance pattern of *H. pylori* isolates is of great importance in the prospect of treatment. The current study aimed to assess the antibiotic resistance patterns as well as the frequency of *cagA* and *vacA* genes in *H. pylori* isolates collected from clinical centers in Tabriz city, Iran.

## Main text

### Methods

#### Sample collection

This cross-sectional study was conducted on 221 gastric biopsies of patients who were referred to clinics and endoscopy centers located in Tabriz city from April 2013 to June 2015. The Cochran's formula was used to determine the sample size. Patients (aged 15–90 years old) had several clinical symptoms, comprising abdominal pain, stomach sore, burping, flatulence, nausea, and vomiting. Exclusion criteria were as follows: non-cooperative patients who refused to give their consent or to participate in the study, patients with a history of *H. pylori* eradication treatment, and those with a history of consumption of antibiotics or proton pump inhibitory drugs within the last 1 month. The endoscopy was performed to obtain gastric biopsy samples. The collection of samples was carried out aseptically from the site of lesion or gastric antrum. After transferring in Stuart transport culture medium (Liofilchem, Italy), all the samples were analyzed in the microbiology laboratory within 4 h of preparation.

#### Culture of samples

The whole samples were cultured on selective Brucella agar (Liofilchem, Italy) plates, containing 5% sterile sheep blood, vancomycin (5 mg/L), trimethoprim (5 mg/L), and polymyxin B (2500 U/L). The plates were incubated under microaerophilic conditions (5% O_2_, 10% CO_2_, 85% N_2_) using the Anoxomat^®^ jar system (USA) at 37 °C for 7 days. Isolates exhibiting Gram-negative curved rods, positive reaction for catalase, oxidase, and urease tests were considered as *H. pylori*. Confirmed isolates were suspended in Eppendorf tubes, comprising of brain heart infusion (BHI) broth supplemented with 5% horse serum and 20% glycerol. The tubes were stored at − 80 °C until future use [15]. A standard control strain, NCTC 11638, was used to confirm the identity of the isolates and to affirm antimicrobial assays.

#### Determination of antibiotic resistance

The antibiotic resistance pattern of the isolates was determined by the disc diffusion method according to the Clinical and Laboratory Standards Institute (CLSI) guidelines [16]. The antibiotics used included clarithromycin (15 µg), metronidazole (5 µg), amoxicillin (25 µg), tetracycline (30 µg), ciprofloxacin (5 µg), and furazolidone (100 µg), which were all procured from Mast Diagnostics Mast group Ltd., Merseyside, UK. After 2–3 days of incubation under microaerophilic conditions, the diameters of the growth inhibition zones were measured in millimeters.

#### DNA extraction and detection of *vacA* and *cagA* genes

For the molecular identification of *vacA* and *cagA* genes, DNA of all the isolates was extracted using a commercial extraction kit (Invitek Molecular, Berlin, Germany) according to the manufacturer’s instructions. The multiplex polymerase chain reaction (M-PCR) for the mentioned genes was conducted in a total volume of 25 µL using the primers listed in Table 1 [17]. For this purpose, 5.5 µL of PCR master mix (5×), consisting of Taq DNA polymerase (0.05 U/µL), MgCl_2_ (3 mM), dNTPs (0.4 mM), was admixed with 1 µL of each primer, 1 µL of template DNA, and 10.5 µL of distilled water. The temperature program was set to an initial denaturation at 94 °C for 4 min, followed by 35 cycles of denaturation at 94 °C for 45 s, annealing at 60 °C for 30 s, extension at 72 °C for 90 s, and the final extension at 72 °C for 4 min. The M-PCR products were separated by electrophoresis on 1% agarose gel and stained in a tank containing ethidium bromide for 15 min. The results were visualized under a UV light using a gel documentation system. *H. pylori* ATCC 43504 and *Salmonella typhimurium* ATCC 14028 strains were used as positive and negative controls, respectively.

**Table 1:** The primer sequences used for the identification of *vacA* and *cagA* genes in *H. pylori* isolates

Gene	Primer sequence (5ʹ → 3ʹ)	Product size (bp)
*vacA*	F-ATGGAAATACAACAAACACAC R-CTGCTTGAATGCGCCAAAC	259
*cagA*	F-GATAACAGGCAAGCTTTTGA R-CTGCAAAAGATTGTTTGGCA	499

#### Statistical analysis

The descriptive statistical tests (numbers and percentages) were performed using SPSS version 20 (IBM^®^, New York, NY, USA) for the frequency rates of antibiotic resistance of *vacA*, and *cagA* gene. Fisher’s exact test was applied to compare the variables, and a *p*-value of less than 0.05 was deemed statistically important.

### Results

Among 221 collected samples, 129 (58.37%) and 92 (41.63%) samples were from males and females, respectively. The mean age of the patients was 36.4 ± 29.7 years old. The culture method detected 100 (45.25%) *H. pylori* isolates from 221 biopsy samples. No statistically significant difference was detected in the distribution of *H. pylori* between different gender and age groups (*p* > 0.05). The highest resistance of the isolates was against metronidazole (79%), followed by amoxicillin (36%), clarithromycin (25%), furazolidone (20%), ciprofloxacin (10%), and tetracycline (8%). Besides, there was no statistically significant association of resistance to different antibiotics with gender and age (*p* > 0.05). Based on the molecular analysis, the frequencies of the *cagA* and *vacA* genes were 63% and 81%, respectively, and difference in the distribution of the two genes between different age and gender groups (*p* > 0.05) was insignificant.

### Discussion

This study demonstrated the prevalence rate of 45.25% for *H. pylori* isolates in Tabriz, the most heavily developed town in the northwest of Iran. However, Navidifar et al. [15] reported a lower rate of prevalence (22.12%) in Yazd, Iran. The present study investigated the resistance rates of *H. pylori* isolates against six antibiotics, and the results indicated a high percentage (79%) of resistance to metronidazole among *H. pylori* isolates. Metronidazole is a prodrug activated by oxygen-insensitive nitroreductases in the bacterial cell, and the well-characterized mechanism of resistance to this antibiotic is mutations in the *rdxA* gene encoding RdxA [18]. Similar to our study, a number of other surveys have reported high resistance rates, ranging from 60 to 77.8%, to metronidazole among *H. pylori* isolates [15, 19–21]. In addition to *H. pylori* treatment, metronidazole is recommended for treating other infections such as parasitic, periodontal, and gynecological infections; the primary resistance to this antibiotic among *H. pylori* isolates may be due to the frequent use of this drug for treating the above-mentioned infections [15, 18].

In this study, there was no correlation (*p* > 0.05) between metronidazole resistance and female gender, as reported by other researchers [15, 22]; however, some others [23–25] explored a positive correlation between metronidazole resistance and female gender [23–25]. The reason for such relationship could be explained by the fact that metronidazole is frequently administered for treating gynecological infections. Following metronidazole, amoxicillin showed the highest percentage of resistance. A mutation in the *pbp-1a* gene has been found as a primary cause of bacterial resistance to amoxicillin [18]. In support of our results, only a few Iranian researchers have reported the relatively high rates of resistance to amoxicillin [26, 27], while several researchers opposed to this result and implied low resistance rates to this antibiotic [15, 28, 29]. Overall, the general resistance to amoxicillin is low worldwide, but owing to the disproportionate use of amoxicillin, particularly for treating common respiratory tract infections, bacteria can develop some mechanisms of resistance to this antibiotic [18].

Our results reflected that about 25% of *H. pylori* isolates were resistant to clarithromycin, an essential component of standard triple therapy for *H. pylori* and a member of macrolide family [30]. The main mechanism of action of macrolides is the inhibition of protein synthesis-dependent RNA by binding to receptors located in the *23S rRNA* gene. Hence, the mutations in this region have been associated with the resistance of *H. pylori* to clarithromycin [31]. The resistance rate to this antibiotic in other regions of Iran has also been reported to be 14.6% to 53.4% [15, 27–29, 32, 33]. Nonetheless, due to the prolonged use of clarithromycin to treat other infectious diseases, which causes the expansion of resistant strains as a result of selection pressure, the effective treatment of *H. pylori* in regimes containing this antibiotic is decreasing [34]. Likewise, around 20% of *H. pylori* isolates were furazolidone-resistant. This antibiotic is a synthetic nitrofuran with a broad spectrum of antimicrobial activities widely recommended for treating bacterial and protozoal infectious diseases. However, some concerns recently arose from using furazolidone because of its potential carcinogenic effect [35]. Mutations occurring in the *2-oxoglutarate acceptor oxidoreductase* (*oorD*) and *pyruvate oxidoreductase* (*porD*) genes are possibly related to the resistance to this antibiotic [36]. The resistance rates of furazolidone in other regions of Iran have mostly been reported to be different, ranging from 4.5 to 61.4% [27, 32, 37], while in some other regions, there was no furazolidone-resistant isolate [15, 38]. One of the main reasons for the emergence of resistance could be the use of furazolidone for the treatment of bacterial and protozoal infections [35].

In the current study, the lowest levels of resistance were shown against tetracycline and ciprofloxacin. The average percentage of the tetracycline resistance rate in Iran is 12.2%. In virtue of the selection pressure, resistance to tetracycline, the same as other antibiotics, is enhanced with the use of this drug [18]. In our study, the resistance rate of *H. pylori* isolates against tetracycline was 8%; however, other studies conducted in Iran have indicated varied rates (from 4.4 to 76.2%) against this antibiotic [15, 26, 32]. Besides, the resistance to ciprofloxacin was detected in 10% of the isolates. The resistance to ciprofloxacin has been studied in other research works, but was less than the rate stated in our study [15, 27, 32].

As mentioned above, the *cagA* and *vacA* genes are the most important determinants of gastric pathogenesis. The frequencies of the *cagA* and *vacA* genes were 63% and 81%, respectively. In previous studies from Iran, Molaei et al. [39], Doosti et al. [40], and Doraghi et al. [41] reported the frequency of 78.6%, 83.5%, and 82.2% for the *cagA* gene in *H. pylori* isolates, respectively, indicating a higher frequency than our study. The prevalence of *vacA* gene in this study was also lower than studies conducted in South Africa by Idowu et al. [42] and in India by Vadivel et al. [43] who reported the prevalence rates of 90.6% and 85.5% in *H. pylori* isolates, respectively. In another study by El Khadir et al. [44] in Morocco, *vacA* and *cagA* genes were detected in 99.5% (638/641) and 61.2% (392/641) of biopsies, respectively. However, Akeel et al. [45] reported the *cagA* gene in 49.2% (63/128) and the *vacA* gene in 100% (128/128) of Saudi patients.

## Conclusion

This study showed the most resistance rates of *H. pylori* isolates against metronidazole, amoxicillin, and clarithromycin. Therefore, using such antibiotics require to be used with more caution. The present study also found a relatively high frequency rate of two main virulence genes (*cagA* and *vacA*) in *H. pylori* strains collected from the northwest of Iran. Moreover, there was no significant association between the prevalence of the two genes in different age and gender groups.

## Limitation

In this study, the minimum inhibitory concentration (MIC) of antibiotics and also the association of different risk factors with *H. pylori* infection were not evaluated. Additionally, the genotyping of *cagA* and *vacA* genes was not performed.

## Data Availability

The data of the current study are available from the corresponding author on reasonable request.

## Abbreviations

CLSI: Clinical and Laboratory Standards Institute
ESBL: Extended spectrum β-lactamases
*cagA*: Cytotoxin-associated gene A
MALT: Mucosa associated lymphoid tissue
MIC: Minimum inhibitory concentration
M-PCR: Multiplex polymerase chain reaction
*oorD*: Oxoglutarate acceptor oxidoreductase
*porD*: Pyruvate oxidoreductase
*vacA*: Vacuolating cytotoxin A
WHO: World Health Organization
